# The Trajectory of Pseudoneglect in Adults: A Systematic Review

**DOI:** 10.1007/s11065-018-9392-6

**Published:** 2018-11-21

**Authors:** Trista E. Friedrich, Paulette V. Hunter, Lorin J. Elias

**Affiliations:** 1 Department of Psychology, University of Saskatchewan, 9 Campus Drive, Saskatoon, SK S7N 5A5 Canada; 2 St. Thomas More College, University of Saskatchewan, 1437 College Drive, Saskatoon, SK S7N 5A5 Canada

**Keywords:** Pseudoneglect, Perceptual Bias, Aging, Older adults

## Abstract

Neurologically healthy adults tend to display a reliable leftward perceptual bias during visuospatial tasks, a phenomenon known as pseudoneglect. However, the phenomenon in older adults is not well understood, and a synthesis of research that examines pseudoneglect using the line bisection task, as well as other tasks, in the context of aging is lacking. The aim of the current systematic review is to integrate the available research on pseudoneglect in late adulthood, and to discuss the association between age and a bias to the left hemispace. The systematic search revealed that five different tasks have been used to examine pseudoneglect in younger and older adults, and that participants over 60 years of age have demonstrated inconsistent perceptual biases (e.g., enhanced leftward bias, suppressed leftward bias, and rightward bias). Based on current evidence, although some age-related trends in the perceptual bias can be identified within each task, no firm conclusions about the effects of age on pseudoneglect can be drawn. A number of recommendations for future research are outlined throughout the review, including use of smaller age ranges within age groups, differentiating between neurologically healthy participants and those with clinical diagnoses, continued examination of gender, and consistent use of stimuli and methods of analyses within each task to improve internal comparability.

Pseudoneglect, a phenomenon first identified by Bowers and Heilman (1980), initially referred to a directional error to the left when neurologically healthy individuals attempted to locate the midpoint of a tactile stimulus – a balsa stick. Since 1980, young neurologically healthy individuals have also been found to err to the left when asked to complete other simple perceptual tasks using different modalities. These tasks have included the line bisection, tactile rod bisection, landmark, greyscales, and lateralized visual detection tasks. During such tasks, participants typically systematically misplace the transection to the left of the objective center, perceive equally bisected lines as longer on the left, or select the greyscale stimulus that displayed the darker end on the left. Although these findings are robust and have been supported by research using animals (Diekamp et al. 2005; Regolin 2006), the magnitude of the bias is small, particularly in comparison to larger errors made by patients experiencing hemispatial neglect.

The modesty of the phenomenon led researchers to question whether leftward errors and biases were an artifact related to random sampling errors in small sample sizes. However, the most recent meta-analysis that integrated peer-reviewed literature examining pseudoneglect and moderating variables in line bisection performance supported the notion that the phenomenon exists (Jewell and McCourt 2000). The meta-analysis consisted of 73 studies and 2119 participants. Jewell and McCourt (2000) reported a leftward bisection bias with effect sizes (*d*) ranging from −.37 to −.44, which was modulated by both task and participant variables. Task variables consisted of modality specific effects (e.g., visual, pointing, tactile, kinesthetic), line length, line position, cueing, direction of scanning, and hand used to complete the task (e.g., left, right, or both hands). Participant variables included age, sex, and handedness.

Of the participant variables examined in this meta-analysis, age is of particular interest. The meta-analysis included a limited number of studies that had examined age-related changes in pseudoneglect, and the conclusion reported may have been premature (Jewell and McCourt 2000). Jewell and McCourt (2000) concluded that younger subjects (less than 40 years-of-age) typically err to the left of center on the line bisection task, whereas older adults (greater than 50 years-of-age) err to the right of center. This conclusion was formulated based on two experiments that specifically focused on age-related changes of pseudoneglect in adults. Since Jewell and McCourt’s (2000) meta-analysis and over the past two decades, researchers have begun investigating the effects of age-related differences in pseudoneglect using a variety of tasks. However, the phenomenon in older adults is not well understood and a synthesis of research that has examined pseudoneglect using the line bisection task, as well as other tasks, in the context of aging is lacking.

Tasks that are used to examine pseudoneglect are also used to assess hemispatial neglect in clinical populations, and research examining populations with neglect often used control subjects who are matched in age to patients who are typically in or beyond their fifth decade of life. Understanding the normal variability demonstrated on visuospatial tasks by older adults can assist researchers and clinicians in interpreting the findings observed in clinical populations (e.g., patients with hemispatial neglect). The aim of the current systematic review is to integrate the available research on pseudoneglect in late adulthood to discuss the association between age and a bias to the left hemispace. Synthesizing the literature on age effects will contribute to an understanding of the normal variability in pseudoneglect demonstrated by neurologically healthy older adults.

## Methods

The systematic review has been conducted in accordance with the PRISMA guidelines (Moher et al. 2015). A university librarian provided general training on search methods, including: (1) review frameworks, (2) choosing databases, and (3) identifying relevant search terms. A review protocol was written but not registered. A second university librarian (1) reviewed the search methods, (2) provided feedback, and (3) assisted in identifying electronic programs to organize the retrieved the articles (e.g., reference management software).

### Search Strategy and Information Sources

To identify relevant literature, DiCenso et al.’s (2005) “population and situation” (PS) framework was used. The PS framework allows the research question to be separated into two key elements: 1) concern for the population (“P”) of interest, in this case, older adults, and 2) the situation (“S”), also called the condition, in this case, pseudoneglect. Synonyms of “older adult” and “pseudoneglect” were used as key words and subject headings to guide a systematic search for relevant literature (see Appendix A for full search specifications, including other search terms). The following databases were searched most recently on 5 July 2017: PsychInfo (1806 to June week 2 2017), Medline (1946 to June week 2 2017), Embase (1947 to June 19, 2017), Web of Science (1900 to July 4, 2017), Scopus (without time restrictions), OpenGrey (1997 to July 5, 2017), and Open Science Framework (January 2013 to July 2017). A search was specifically conducted in the OpenGrey database and Open Science Framework repository, and grey literature was included in the review to minimize publication bias. In addition to systematic electronic searches in the above databases, reference lists of reviews and retrieved articles were searched for additional studies (i.e., searching backwards), and citation searches on key articles were performed (i.e., searching forwards).

### Eligibility Criteria

The review focused on integrating literature that examined pseudoneglect in relation to aging using a variety of tasks. An inclusive inclusion criterion was used to screen for articles to allow for a broader overview of the literature. Studies were eligible if they included one of the following tasks to examine pseudoneglect: line bisection task, landmark task, greyscales task, grating scales task, tactile rod bisection task, or lateralized visual detection task, and if they included participants who were 60 years of age or older and identified as neurologically healthy (i.e., not having a diagnosis of a neurological condition). Results were not restricted by study type, date of publication, or gender, but were limited to original research articles written in English.

After each database search was conducted, results were compiled using EndNote X8 and screened using the reference manager Rayyan. Duplicate records of identical studies were initially removed using EndNote and subsequently (i.e., if any were missed) using Rayyan. Titles and abstracts were screened by TF in Rayyan and records not fulfilling the inclusion criteria were excluded. Subsequently, the articles that were marked as potentially relevant were examined for eligibility by reviewing the full-text. Descriptive information from each study, including the aim of the study and the study population (e.g., sample size, age, gender), was extracted into Tables 1, 2, 3, 4 and 5 (as suggested by Green et al. 2006).

Previous research has reported low inter-task reliability and correlations between visuospatial tasks that examine pseudoneglect (Learmonth et al. 2015a; Rueckert et al. 2002). This suggests that pseudoneglect may be multi-component phenomenon and subject to variations based on task demands (Learmonth et al. 2015a). If this is the case, it raises the possibility that the tasks are also subject to different patterns of age-related changes (Benwell et al. 2014; Brooks et al. 2011; Friedrich et al. 2016; Fujii et al. 1995; Fukatsu et al. 1990; Schmitz and Peigneux 2011; Varnava and Halligan 2007). As a result, the tasks used to examine pseudoneglect in this systematic review were categorized and examined independently in an attempt to minimize variability and inconsistency in the results.

## Results

### Study Selection

**Fig. 1:**
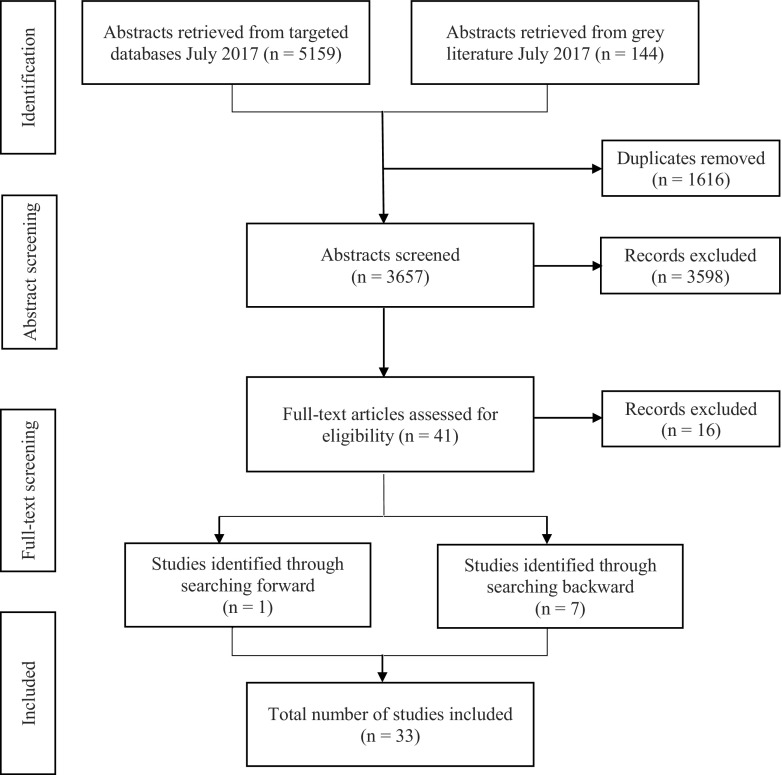
An outline of the search process using the Preferred Reporting Items for Systematic Reviews and Meta-Analyses (PRISMA) diagram (Moher et al. 2015)

The database search generated 5196 titles of which 1616 were duplicates, resulting in a total of 3657 unique articles (see Fig. 1). After title and abstract screening, 3598 articles did not meet eligibility criteria and were therefore excluded. Of the excluded articles, the majority failed to meet inclusion criteria either because the task under study was not relevant (e.g., assessed memory or language) or because the study population did not include older adults. Using the same criteria, the full-text of the remaining 41 titles were assessed for eligibility. At this point, four additional articles were excluded as they employed neuropsychological test batteries designed to assess hemispatial neglect but did not include tasks specifically designed to assess pseudoneglect (e.g., cancellation, copying, and personal neglect tasks). Although such tasks are commonly used to identify hemispatial neglect, healthy participants often display ceiling effects on the tasks (Schindler et al. 2006); thus, they are not typically used to examine pseudoneglect and the results were not considered for the review. Another eight articles employed the chimeric faces task, a task that requires judgment of similarity, gender, age, attractiveness, or emotional expression of a constructed image where the left and right sides differ, were excluded. During the chimeric faces task, participants typically demonstrate a left perceptual bias (i.e., a predisposition to base decisions on the left side) for chimeric images; however, the bias has been proposed to result from right hemisphere dominance for face processing rather than right hemisphere dominance for visuospatial attention processing; thus, results are not considered relevant to this review. A further four articles were excluded because they examined adults younger than 60 years of age. This reduced the pool of articles to 25. However, seven additional articles were identified through backwards searching, and one dissertation was identified through forward searching. Thus, in total, 33 titles qualified for inclusion.

The 33 titles were published between 1989 and 2017 and consisted of journal articles, theses, and conference presentations. Five tasks were used to examine pseudoneglect: the line bisection task, landmark task, greyscales task, tactile rod bisection task, and lateralized visual detection task. Specifically, the line bisection task requires participants to place a mark with a pencil or cursor through the center of a horizontal line to divide the line in equal halves (Albert 1973). Similarly, the tactile rod bisection task requires participants, with their eyes closed, to place their index finger at the perceived middle of a wooden doweling rod after exploring the entire length of the rod (Brooks et al. 2011). A non-manual variant of the line bisection task is the landmark task, which requires the participant to make a two-alternative forced choice decision regarding the length of the two halves of a line that is pre-bisected in the center (Milner et al. 1992). In contrast to judgments in size required by the line bisection and landmark task, the greyscales task requires judgments in luminance. The task requires participants to judge which of two horizontal mirror-imaged equiluminant gradients (i.e., one shaded from black to white and the other shaded from white to black) appears darker (Nicholls et al. 1999). Last, the lateralized visual detection task requires participants to detect small dots that briefly appear at the individual’s peri-threshold in the left or right side of space and detection accuracy is calculated (Hilgetag et al. 2001). In total, 21 titles employed the line bisection task, six employed the landmark task, one employed the line bisection and landmark task, two employed the greyscales task, two employed the tactile employed the tactile rod bisection task, one employed the lateralized visual detection task, and one employed five perceptual tasks (line bisection task, landmark task, grating scale task, greyscales task, and lateralized visual detection).

### Line Bisection Task

Of the studies included in the systematic review, 23 examined how performance on the line bisection task varied with age. There was considerable variability in the results, with 14 studies pointing to an attenuated leftward bias with age, one study pointing to a leftward bias, two studies pointing to enhanced bias, and six studies not finding any significant age-dependent effects (see Table 1). For example, with respect to attenuated leftward biases, Fujii et al. (1995) compared three groups of 36 participants to investigate the effect of age on the line bisection task. The oldest age group (61–82 years of age) bisected lines to the right of center, and their bisections were also significantly more rightward than the middle (42–60 years of age) and young (21–30 years of age) groups, who were accurate in their bisections and did not deviate from center. Similarly, Barrett and Craver-Lemley (2008) reported younger adults demonstrated a leftward line bisection bias that was significantly larger than the accurate bisection demonstrated by the older participants. Others have found similar results (Barrett and Craver-Lemley 2008; Chen et al. 2011; Failla et al. 2003; Goedert et al. 2010).

**Table 1:**
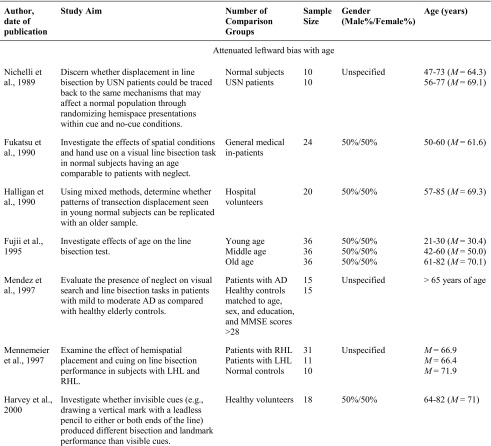

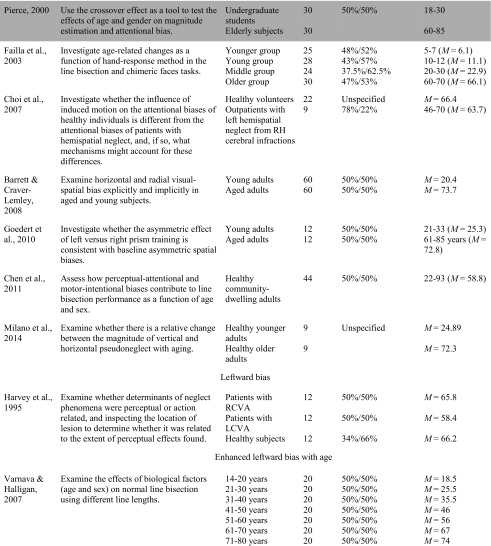

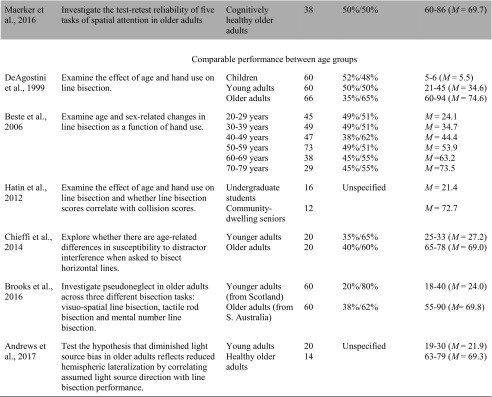
Characteristics of included studies examining the line bisection task

The studies highlighted are considered grey literature (e.g., theses and poster presentations) and were not retrieved from peer-reviewed journals. Terms used to describe age groups are as reported in the original studies.

*USN* Unilateral Spatial Neglect; *AD* Alzheimer’s Disease; *MMSE* Mini Mental State Exam; *LCVA* left hemisphere stroke; *LHL* Left Hemisphere Lesion; *RCVA* right hemisphere stroke; *RHL* Right Hemisphere Lesion; *Crossover Effect* neurologically healthy participants err to the left when bisecting medium and long lines, but err rightward when bisecting short lines

In contrast, a single published study reported evidence of an enhanced leftward bias with age, particularly for participants who were women. Varnava and Halligan (2007) examined the effects of age and sex on line bisection by examining seven even age cohorts using three different line lengths. Age and sex-related differences were found in bisecting different line lengths with women over 30 demonstrated larger leftward deviations as line length increased, whereas women under 30 had similar leftward deviations on all line lengths. In contrast, men, as a group, did not demonstrate a trend and deviated to the left on short lines and either to the left or right on longer lines. Furthermore, DeAgostini, Curt, Tzortzis, and Dellatolas (1999) failed to find significant difference in the magnitude and direction of the bisection deviation between children, adults, and older adults, and a similar finding of insignificant differences between age groups have also been observed by others (Andrews et al. 2017; Beste et al. 2006; Brooks et al. 2016; Chieffi et al. 2014; Hatin et al. 2012).

When such variability in results exists, it becomes important to more precisely examine research questions and methods for indications of the potential causes of these discrepancies, beyond age and task. For example, one important observation from this review is that researchers have used different comparison groups to assess age-related effects on line bisection. Some have compared the performance of healthy older adults to patients with hemispatial neglect (Choi et al. 2007; Mennemeier et al. 1997; Nichelli et al. 1989) or Alzheimer’s Disease (Mendez et al. 1997), whereas others have compared the performance of participants in various age groups. Further, researchers who have compared performance of participants in various age groups have used different age categories and have varied in the number of age categories examined. For example, some researchers limited comparisons between a single younger and older age group (Andrews et al. 2017; Barrett and Craver-Lemley 2008; Brooks et al. 2016; Chieffi et al. 2014; Goedert et al. 2010; Hatin et al. 2012; Milano et al. 2014; Pierce 2000), whereas others compared multiple age groups (De Agostini et al. 1999; Failla et al. 2003; Fujii et al. 1995), some of which were evenly divided across the adult lifespan (Beste et al. 2006; Varnava and Halligan 2007). Furthermore, other researchers did not use a comparison group and limited the population examined in their research to older adults who had comparable age to patients with neglect (Fukatsu et al. 1990; Halligan et al. 1990; Maerker et al. 2016).

In addition to comparing older adults to various age groups, studies also differed in their approach to define healthy older adults and accounting for cognitive impairment. Differentiating between neurologically healthy participants and participants who have symptoms of cognitive impairment assists in establishing whether age-related differences in perceptual biases are due to neuropathology (Learmonth et al. 2017). Of the 23 studies, five studies used the Mini Mental State Exam to screen for symptoms of mild cognitive impairment in older participants. When cognitive performance was assessed, older adults who were identified as neurologically healthy demonstrated accurate bisections (Barrett and Craver-Lemley 2008; Chieffi et al. 2014; Mendez et al. 1997) or demonstrated a leftward bias that did not differ from younger adults (Brooks et al. 2016; Chen et al. 2011 (female participants)), except in the case of a study by Chen et al. (2011), who identified an interaction between sex and age with only male participants demonstrating a rightward bisection bias with age.

Another observation is that studies did not universally examine gender differences in age-related differences on bisection performance. More specifically, some researchers did not specify the gender of participants (Andrews et al. 2017; Choi et al. 2007; Hatin et al. 2012; Mendez et al. 1997; Mennemeier et al. 1997; Milano et al. 2014; Nichelli et al. 1989), whereas others specified and examined gender-related effects on line bisection performance (Barrett and Craver-Lemley 2008; Beste et al. 2006; Chen et al. 2011; Pierce 2000; Varnava and Halligan 2007). In these studies, an interaction between sex and age was common, with men demonstrating an attenuated leftward bias or rightward bias with age, and women demonstrating a leftward bias that was either comparable to that shown by younger adults (Barrett and Craver-Lemley 2008; Chen et al. 2011; Pierce 2000), or larger than that of younger adults (Varnava and Halligan 2007).

In addition to variation in the participant variables examined, it was observed that researchers also used different methods to bisect lines, including variations in the hand used to make bisections. Five researchers specifically investigated age-related differences in line bisection as a function of hand use (Beste et al. 2006; De Agostini et al. 1999; Failla et al. 2003; Fukatsu et al. 1990; Hatin et al. 2012). The results were largely inconsistent. For example, Fukatsu et al. (1990) found that participants in their fifties and sixties demonstrated deviations significantly to the right of center when bisections were conducted with the right hand and accurate bisections (i.e., not significantly different from zero) when using the left hand, whereas Hatin et al. (2012) found that older adults were accurate when using their right hand and that bisections were significantly to the left of true center when completing the task with their left hand. Further, Failla et al. (2003) and De Agostini et al. (1999) both identified a significant interaction between hand used and age group. Failla et al. (2003) reported that the interaction was driven by a stronger and consistent leftward bias with left hand across age groups than when using the right hand and both hands (i.e., bimanual). Specifically, the oldest age group demonstrated a significant deviation to the right of true center when using the right hand and demonstrated accurate bisections when using both hands. Whereas De Agostini et al. (1999) reported that all age groups demonstrated a significant constant bias to the left of center when using the left and right hand, except for male children and male older adults when using the right hand who demonstrated accurate bisections. An interaction between hand use, age, and sex was also reported by Beste et al. (2006), as the findings in their study revealed hand-use differences in women for the first three decades of life (i.e., a leftward bias when using the left hand and a rightward bias when using the right hand), which disappeared in 50 and 60-year-olds and re-emerged in their 70-year-olds. This was in contrast to men, who, in all age groups, demonstrated leftward bisections when using the left hand and accurate bisections when using the right hand (Beste et al. 2006).

In reviewing the studies retrieved it was also observed that the line bisection task differed with regard to stimulus properties, such as line length. Of the studies reviewed, stimulus line length varied from 20 to 400 mm in length and the number of different lengths presented to participants varied from two to 13. Of the studies reviewed, only two explicitly examined the effects of age on line bisection using different line lengths. Both studies reported an interaction between age and line length, but the findings were contradictory (Pierce 2000; Varnava and Halligan 2007). Pierce (2000) reported that younger adults bisected to the left of true center on short lines and to the right of true center on longer lines (i.e., the crossover effect), whereas older adults bisected to the right of true center on short lines and became more accurate as line length increased. In contrast, Varanava and Halligan (2007) reported that four older age cohorts (31–80 years) deviated to the left of true center with greater magnitude on the longest line compared to the two shorter lines, whereas the two youngest cohorts (14–30 years) deviated to a similar magnitude across all three line lengths.

Together, the variation in methods (e.g., hand used) and stimulus properties (e.g., line length) employed within these studies, as well as the use of different comparison groups and variability in accounting for gender and cognitive impairment, makes it difficult to assess the degree to which differences in performance on the line bisection task is influenced by age. Nevertheless, some general patterns in the relationship between bisection performance and aging were apparent. For instance, researchers using the line bisection task commonly reported an attenuation of the leftward bias with age, with older adults demonstrating bisections further to the right than younger adults (Barrett and Craver-Lemley 2008; Chen et al. 2011; Failla et al. 2003; Fujii et al. 1995; Goedert et al. 2010; Harvey et al. 2000; Milano et al. 2014; Pierce 2000). Similarly, researchers also reported that older adults, as a control group, demonstrated accurate bisections (Choi et al. 2007; Harvey et al. 2000; Halligan et al. 1990; Mendez et al. 1997; Nichelli et al. 1989) or bisections to the right of true center (Fukatsu et al. 1990; Mennemeier et al. 1997). However, these results were not universal. In a number of studies, there were no differences between older and younger age groups with all age groups either bisecting lines to the left of true center (Andrews et al. 2017; Beste et al. 2006; Brooks et al. 2016; De Agostini et al. 1999; Hatin et al. 2012) or demonstrating accurate bisections (Chieffi et al. 2014). Further, a limited number of studies reported older adults demonstrating a stronger leftward bias compared to younger adults (Varnava and Halligan 2007), or a bias to the left of true center without comparing performance to another age group (Choi et al. 2007; Harvey et al. 1995; Maerker et al. 2016).

### Landmark Task

Considerable variability in the direction of the perceptual bias was also identified when researchers examined pseudoneglect in older adults using the landmark task. Of the nine studies that examined how performance on the landmark task varied with age, five studies supported an attenuated leftward bias with age, two studies supported a leftward bias, and two studies did not find any significant age-related effects (see Table 2). For example, with respect to an attenuated leftward bias with age, Schmitz and Peigneux (2011) reported differences in response patterns between elderly and younger adult participants. Older adults did not demonstrate a bias and judged evenly bisected lines at chance level (i.e., selected the left section of the line as longer/right as shorter for half of the trials), whereas younger participants judged the left end of the lines as longer at an above chance level, thus demonstrating a leftward bias. Similarly, Benwell et al. (2014) examined the effect of age on lateralized visuospatial bias during the landmark task using three different line lengths. Overall, younger and older adults identified different subjective midpoints with younger adults perceiving the midpoint significantly more to the left compared to older participants. Others have also found an attenuated leftward bias with age (Learmonth et al. 2015b; Harvey et al. 2000; Maerker et al. 2016; Schmitz et al. 2013). Nevertheless, this finding is not universal. Harvey, Poll, Roberson, and Olk (2000) investigated the effect of visible and invisible cues on the landmark task using asymmetrically transected stimuli in a group of 18 older adults. In the no-cue condition, the older adults demonstrated significantly more leftward responses compared to chance. Similar results in a no-cue condition were reported by Harvey et al. (1995) when examining healthy older adults. Furthermore, researchers using the landmark task have also failed to identify age-related differences (Learmonth et al. 2017), even when controlling for participants’ race, education, total weighted occupational prestige, visual acuity, and WSIC-IV Information scale score (McPherron 2015). For example, Learmonth et al. (2017) analyses of the behavioural responses on the landmark task indicated that participants were accurate in their judgment of the midpoint with both younger and older adults failing to demonstrate a perceptual bias.

**Table 2:**
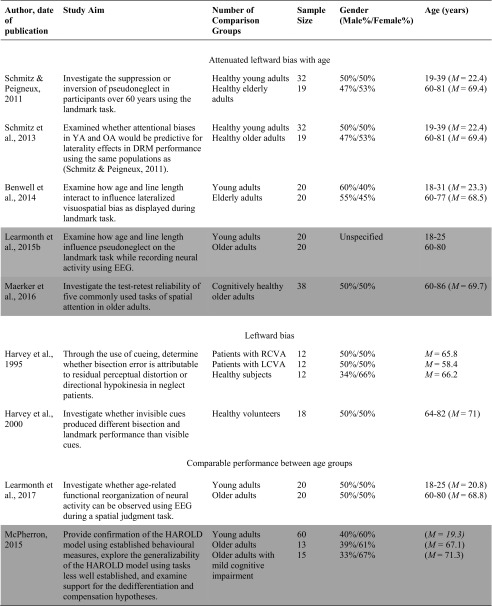
Characteristics of included studies examining the landmark task

The studies highlighted are considered grey literature (e.g., theses and poster presentations) and were not retrieved from peer-reviewed journals. The terms used to describe the age groups are consistent with the terms used by authors.

*YA* Young adults; *OA* Older adults; *DRM* Deese-Roediger-McDermott paradigm

Given the variability in reported findings, it is important to note that researchers diverged in their choice of dependent variable and in how biases were calculated. For example, some researchers calculated a leftward perceptual bias as the percentage of left longer/right shorter responses for evenly bisected lines (Schmitz and Peigneux 2011; Schmitz et al. 2013), others calculated a leftward response based on the number of leftward choices participants made when asked which end of line the they thought the transection was closest to (Harvey et al. 1995; Harvey et al. 2000), and still others used a cumulative logistic psychometric function (i.e., a measure of the precision of midpoint judgments) and a point of subjective equality (i.e., the perceived midpoint of the line) for unevenly bisected lines (Benwell et al. 2014; Learmonth et al. 2017).

### Greyscales Task

A limited number of studies included in the systematic review examined age-related differences in pseudoneglect using the greyscales task. In contrast to the variability in results when researchers have employed the line bisection and landmark task, research using the greyscales task has generated fairly consistent results (see Table 3). The two journal articles published in peer-reviewed journals retrieved in the systematic search both reported that older adults demonstrated a significant leftward bias on the greyscales task. For example, Mattingley et al. (2004) compared the performance of neurologically healthy older adults to a sample of patients with a unilateral stroke on the greyscales task found that healthy older adults demonstrated a small leftward bias. Similarly, Friedrich et al. (2016) compared the performance of seven age groups on the greyscales task and each age group judged the mirrored equiluminant stimulus as darker when the stimulus displayed the darker end on the left. A significant difference was also found between the seven age groups with the oldest age group (80–89 year olds) demonstrating a significantly stronger leftward bias compared to the youngest age group (18–29 year olds). Further, a negative relationship was found between age and a leftward bias with the magnitude of the bias increasing with age. In contrast, a poster presented by Maerker et al. (2016) examined the test-retest reliability of tasks of spatial attention in older adults, including the greyscales task. In this study, older adults demonstrated a rightward bias.

**Table 3:**
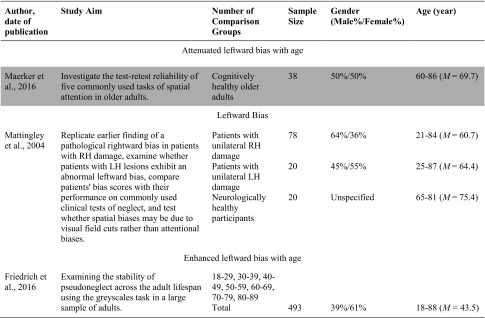
Characteristics of included studies examining the greyscales task

The studies highlighted are considered grey literature (e.g., theses and poster presentations) and were not retrieved from peer-reviewed journals. The terms used to describe the age groups are consistent with the terms used by authors.

*RH* Right Hemisphere; *LH* Left Hemisphere

### Tactile Rod Bisection Task

Of the 32 studies retrieved in the systematic search, two examined the developmental trajectory of pseudoneglect using the tactile rod bisection task. In both studies, Brooks et al. (2011, 2016) reported that older adults demonstrated a leftward bias that was comparable to younger adult participants (see Table 4). For example, when the side from which the bisection started was counterbalanced, Brooks et al. (2011) reported both older (60–96 years) and middle-aged (18–55 years) adults demonstrated a leftward bias (i.e., a negative mean percent deviation score) that was significantly different from zero. It is noteworthy to mention that when comparing the three age groups, including the youngest age group (6–13 years), a trend for a greater leftward bisection bias with age appeared, but fell short of significance. Similarly, Brooks et al. (2016) recruited younger (18–40 years) and older (55–90 years) participants and the mean percent deviation demonstrated by both age groups was leftward and significantly different than zero, and bisection biases did not differ between the two age groups.

**Table 4:** Characteristics of included studies examining the tactile rod bisection task

Author, date of publication	Study Aim	Number of Comparison Groups	Sample Size	Gender (Male%/Female%)	Age (year)
	Comparable performance between age groups				
Brooks et al. 2011 (Experiment 1)	Examine representational forms of pseudoneglect across the lifespan and how performance is mediated by the spatial direction from which the judgement was made.	3–6 years 7–8 years 9–10 years 11–12 years 13–20 years 22–40 years 41–60 years 61–84 years	72 108 95 59 22 86 79 28	Unspecified	*M* = 5.4 *M* = 7.5 *M* = 9.5 *M* = 11.3 *M* = 15.5 *M* = 35.0 *M* = 47.1 *M* = 70.8
Brooks et al. 2011 (Experiment 2)	Examine representational forms of pseudoneglect across the lifespan and how performance is mediated by the spatial direction from which the judgement was made while controlling for gender.	6–13 years 18–55 years 60–96 years	24 24 24	Unspecified	*M =* 9.4 *M* = 30.3 *M =* 74.2
Brooks et al. 2016	Investigate pseudoneglect in older adults across three different bisection tasks: visuospatial line bisection, tactile rod bisection and mental number line bisection.	Younger adults Older adults	60 60	20%/80% 38%/62%	18–40 (*M* = 24.0) 55–90 (*M* = 69.8)

The terms used to describe the age groups are consistent with the terms used by authors

An additional observation reported by Brooks and colleagues in both studies (2011; 2016) was that the side from which the bisection started was crucial for the magnitude of the bias observed. When bisection started from the right side of the rod participants demonstrated a leftward bias, whereas participants demonstrated accurate bisections when beginning from the left side. Interestingly, Brooks et al. (2011) reported that the oldest age group was the most sensitive to starting side compared to the other two age groups, whereas Brooks et al. (2016) found similar start side effects for both the younger and older age group.

### Lateralized Visual Detection Task

Only one published study retrieved in the systematic search examined age-related differences in performance on the lateralized visual detection task (see Table 5). During the task, small squares were presented either to the left of a fixation cross, to the right, or bilaterally for 40 milliseconds, and participant’s ability to accurately detect the stimuli was examined. At baseline, Learmonth et al. (2015b) found that younger adults were more sensitive to detecting stimuli in the left visual field, reflecting a leftward attentional bias, whereas the older adults did not demonstrate a consistent bias and were equally sensitive to detecting stimuli in the left and right visual field.

**Table 5:** Characteristics of included study examining the lateralized visual detection task

Author, date of publication	Study Aim	Number of Comparison Groups	Sample Size	Gender (Male%/Female%)	Age (year)
Learmonth et al. 2015b	Examine whether atDCS would reinstate an adaptive “youth-like” pattern of right hemispheric dominance for spatial attention in older adults.	Young adults Older adults	20 20	45%/55% 50%/50%	18–24 (*M* = 20.9) 60–77 (*M* = 66.6)

The terms used to describe the age groups are consistent with the terms used by authors. atDCS = anodal transcranial direct current stimulation

### Summary of Results

Within similar tasks, the studies included in the review reported inconsistent findings, as well as variability in the methods used. However, as can be seen in Table 6, a number of notable trends appeared within each task included in the review.

**Table 6:** Summary of the different tasks used and the frequency of results that supported an attenuated leftward bias with age, enhanced leftward bias with age, or comparable performance

Task	Attenuated leftward bias with age	Enhanced leftward bias with age	Leftward bias	Comparable performance between age groups
Line Bisection	14	1	1	6
Landmark	5	0	2	2
Greyscales	1	1	1	0
Tactile Rod Bisection	0	0	0	2
Lateralized Visual Detection	1	0	0	0

Studies employing the line bisection task commonly reported an attenuated leftward bias with age (Barrett and Craver-Lemley 2008; Chen et al. 2011; Choi et al. 2007; Failla et al. 2003; Fukatsu et al. 1990; Fujii et al. 1995; Goedert et al. 2010; Halligan et al. 1990; Harvey et al. 2000; Mendez et al. 1997; Mennemeier et al. 1997; Milano et al. 2014; Nichelli et al. 1989; Pierce 2000), with the minority of studies reporting older adults demonstrated a stronger leftward bias (Varnava and Halligan 2007) or no difference in performance between age groups (Andrews et al. 2017; Beste et al. 2006; Brooks et al. 2016; De Agostini et al. 1999; Hatin et al. 2012). Similarly, studies using the landmark task most commonly reported an attenuation of the leftward bias with age (Benwell et al. 2014; Learmonth et al. 2015b; Maerker et al. 2016; Schmitz and Peigneux 2011; Schmitz et al. 2013), but again these results were not universal. A subset of studies did not find a difference in performance between older and younger adults (Learmonth et al. 2017; McPherron 2015), and Harvey et al. (1995, 2000) reported that older adults demonstrated a leftward bias. In contrast, articles published in peer-review journals consistently reported that older adults demonstrated a leftward bias on the greyscales task (Mattingley et al. 2004) and stronger bias compared to younger adults (Friedrich et al. 2016). Consistent findings were also identified in studies using the tactile rod bisection task. Older adults were reported to have demonstrated a leftward bias comparable to younger adults in both studies (Brooks et al. 2011, 2016).

## Discussion

The aim of the systematic review was to aggregate and summarize age-related differences in performance on tasks used to examine pseudoneglect. Following a systematic search for relevant studies, multiple studies were identified that employed the line bisection task, and a smaller number of studies utilized the landmark, greyscales, tactile rod bisection, and lateralized visual detection tasks. Together, the literature retrieved was characterized by inconsistent results and large variability in study design. Unsurprisingly, this conclusion is identical to the finding reported in Jewell and McCourt’s (2000) qualitative review of the line bisection literature. Even when studies employed identical tasks, they varied in methods (e.g., hand used, direction of scanning), stimuli (e.g., stimulus length, number of stimuli viewed), and approach to comparing participants (e.g., gender, handedness), including structure of age groups (e.g., number of age groups, age range within groups). These differences make it difficult to assess the degree to which performance is influenced by age; thus, it is premature to draw conclusions based on the literature included in the review. However, when comparing the identified studies that examined age-related differences in pseudoneglect a number of observations were noteworthy.

All of the studies included in the review used a cross-sectional design. The analysis of cross-sectional samples varying in age is consistent with the research paradigm in gerontology that has been predominately used to understand cognitive aging (Hofer et al. 2002). However, researchers have questioned the utility of using cross-sectional studies to understand age-related changes, and have argued that understanding aging also requires analysis of change within individuals (Hofer et al. 2002). Relying solely on cross-sectional research to understand pseudoneglect across the life span may be misleading and is unlikely to provide an accurate understanding of longitudinal change or the effect of chronological age. Cross-sectional data has been found to provide unreliable estimates of age-related cognitive decline by conflating the effect of age with cohort effects (Singh-Manoux et al. 2012). Further, research examining cognitive aging has commonly reported discrepancies between cross-sectional and longitudinal age trends with between-person cross-sectional comparisons reporting declines in functioning beginning in early adulthood, whereas within-person longitudinal comparisons report stability or increases in cognitive performance (Salthouse 2010). In an area of research that is dominated by cross-sectional associations between age and pseudoneglect, longitudinal data that are adequately powered are essential for drawing conclusions regarding change with chronological age. To fully understand pseudoneglect across adulthood, future research could benefit from basing conclusions on results derived from multiple methods of data collection and analysis (Salthouse 2011).

Further, when examining cross-sectional differences, the age ranges studied varied substantially. The majority of studies included in the review used a modal “extreme age group design” (Marsiske and Margrett 2006, pp. 320) and categorized participants into younger and older adult groups, with the age range within the older adult age group spanning 20 to 30 years (e.g., 60–80 years or older). Comparing extreme groups of younger and older adults is problematic as the variance associated with middle-aged adults is omitted and inflates estimates of age-related differences. Further, because changes in cognitive functioning often occurs continuously across adulthood, results based on lateral biases observed over a large period of older age (e.g., 20–30 years) could be misleading with regard to the origin of age-related differences and whether the identified age relations are linear. Fewer studies included a middle age group, and only three studies included in the review categorized age groups with smaller age ranges (e.g., 10-year cohorts; Beste et al. 2006; Friedrich et al. 2016; Varnava and Halligan 2007). These three studies reported an enhanced leftward bias with age (Friedrich et al. 2016), particularly as demonstrated by women (Varnava and Halligan 2007), or reported comparisons between age groups that did not reach levels of significance (Beste et al. 2006).

The use of broad age categories and cross-sectional design may be contributing to the variability of perceptual biases observed in older adults within and between the various tasks used to examine pseudoneglect, and may be inhibiting researchers’ ability to understand *when* changes occur. Improving design by examining age groups with smaller age ranges, or using longitudinal methods, is critical, as the extent of changes in cognitive performance may vary considerably over a large age range (e.g., 20 to 30-year span) in older age. Research on the course of intellectual abilities, including spatial orientation, over the adult lifespan has revealed that performance plateaus after a peak in young adulthood until the late 50’s or early 60’s, and then declines at a slow pace until the late 70’s, when decline is often accelerated (Schaie 1994). Specifically, research over 35 years (six testing cycles) within the Seattle Longitudinal Study showed that decline in cognitive abilities is not reliably confirmed prior to age 60, and fewer than half of the participants showed reliable decrements at age 74; however, by age 81, most abilities decline by one standard deviation (Schaie 1993, 1994). On this basis, large age ranges spanning from 60 to the late 80’s, as typically seen in studies of pseudoneglect, are likely insufficient, and may be resulting in large within group differences leading to the reporting of central or attenuated leftward biases. Thus, the conclusion that pseudoneglect becomes rightward with age may be invalid. Of the studies included in the review, only Friedrich et al. (2016) categorized participants in 10-year age cohorts and examined adults over 80 years of age as a separate age group. Interestingly, of the seven age groups examined by Friedrich et al. (2016), only the oldest age group (80–89 year olds) demonstrated an asymmetry score that was significantly different from the youngest age group (18–29 year olds).

Further, when studies differ in comparison groups (e.g., comparing a sample of younger adults to a sample of older adults, comparing three or more age groups) and focus on different age ranges, it may not be meaningful to treat the results obtained as equally comparable. For example, differences that have been reported to occur at approximately 60 years of age (mean age of participants was 61.6 years; Fukatsu et al. 1990) may not involve the same mechanisms as age-related differences that have been reported to occur at 75 years of age (mean age of older participants was 74.6 years; De Agostini et al. 1999). Using smaller age ranges within age groups will likely assist researchers in observing age-related differences, and differentiating an age at which there is a reliably detectable change in pseudoneglect. Understanding when age-related differences begin to occur (i.e., mid-life versus very-late life) and examining the specific age groups identified will assist in enhancing the value and relevance of the research.

Furthermore, a consistent categorization of older adults and specification of whether participants are neurologically healthy in the studies reviewed was limited. Large individual difference in cognitive performance and rate of change observed with aging may also be contributing to the variability in findings. The cognitive status of older adults has been found to have extensive heterogeneity and, despite accounting for clinical diagnoses (e.g., normal cognitive function, MCI, dementia), rates of change can vary from a decline of 0.3 SD per year to improvements of 0.1 SD per year (Mungas et al. 2010). The cognitive status of older individuals is complex and influenced by many variables in addition to age, including, but not limited to, brain injury and disease, mental health, health status, and exposure to substances and medications (Mungas et al. 2010). The multiple deleterious and protective factors that influence the variance in cognitive function and rate of change with age could also be influencing the inconsistent findings revealed in this systematic review.

The majority of studies included in the review examined adults over 60 years of age and did not screen for symptoms of neuropathology, such as mild cognitive impairment or dementia. Of the 32 studies, six screened for mild cognitive impairment and examined whether younger and older age groups differed in general cognitive performance. When cognitive performance was assessed, older adults demonstrated accurate bisections (Barrett and Craver-Lemley 2008; Chieffi et al. 2014; Mendez et al. 1997) or demonstrated leftward spatial bias that did not differ from younger adults (Brooks et al. 2016; Chen et al. 2011 (female participants); McPherron 2015), except for Chen et al. (2011) who identified an interaction between sex and age with only male participants demonstrating rightward bisection biases with age. Given that cognitive screening was employed in a limited number of studies included in the systematic review, it is difficult to determine the presence of neuropathology and whether age-related differences are related to *healthy* aging. Future research would benefit from examining lateral biases in healthy older adult populations by incorporating measures that screen for symptoms mild cognitive impairment. Further, it would be useful to compare the lateral biases of older adults with and without symptoms of cognitive impairment to examine the effects of neuropathology on age-related differences in pseudoneglect and understand the continuum of normal and pathological aging. Of the 32 titles included in the systematic review, only two titles compared the performance of older adults with and without symptoms of pathological aging (McPherron 2015; Mendez et a., 1997) and comparisons did not reach statistical significance.

Individual characteristics in addition to age, such as gender (see Jewell and McCourt 2000, for review) have also been investigated to understand the association between gender and lateral perceptual biases. Of specific interest, a number of manual line bisection studies that surfaced in this review reported sex-differences in age-related differences on bisection performance. Age effects appeared to be stronger in males, as men typically demonstrated an attenuated leftward bias or rightward bias, whereas women demonstrated a leftward bias comparable in magnitude to younger participants (Barrett and Craver-Lemley 2008; Chen et al. 2011; Pierce 2000; Varnava and Halligan 2007). However, Beste et al. (2006) reported discrepant results, as men in each age group bisected to the left of true center when using their left hand, whereas women in all age groups bisected to left of true center except for women 50 to 59 years of age. In contrast, tasks that reduce the influence of motor cuing (e.g., landmark and greyscales task) failed to find differences in the magnitude of pseudoneglect between males and females (Benwell et al. 2014; Friedrich et al. 2016; Learmonth et al. 2017; Schmitz and Peigneux 2011; Schmitz et al. 2013). Consistent with a hypothesis proposed by Benwell et al. (2014), gender- specific aging effects may be influenced by non-perceptual factors, such as motor cueing. Future research that accounts, or controls, for stimulus factors and experimental methods will assist in deconstructing gender specific aging effects.

Heterogeneity within tasks with regard to differences in the stimuli used and method used to calculate the dependent variable decreases internal comparability. For example, studies that used the line bisection task differed in the size and the number of stimuli presented to participants, which also influenced the number of trials used to calculate lateral biases. In the studies included in the review, stimulus length varied from 20 to 400 mm in length, the number of different lengths presented varied from two to 13 different lengths, and presentation of the lines varied from presentation of a single line to multiple lines on a page. Studies that used the line bisection task also differed with regard to which hand participants were instructed to use. Some studies specified using the right, left, or both hands (i.e., bimanual), whereas hand use was not specified in other studies. Studies that used similar tasks also differed in how perceptual biases were calculated. For example, studies that used the landmark task calculated a leftward perceptual bias as the percentage of left longer/right shorter responses for evenly bisected lines (Schmitz and Peigneux 2011; Schmitz et al. 2013), others calculated a leftward response based on the number of leftward choices participants made when asked which end of the line they thought the transection was closest to (Harvey et al. 2000), and still others used a cumulative logistic psychometric function (i.e., a measure of the precision of midpoint judgments) and a point of subjective equality (i.e., the perceived midpoint of the line) for unevenly bisected lines (Benwell et al. 2014; Learmonth et al. 2017). Differences in the duration of the stimulus presentation and examination of response time also varied. Stimulus presentation ranged from 150 ms to 1000 ms, and instructions regarding responses varied from no time limits to instructions that emphasized responding as quickly as possible. Such differences in task instructions, administration, and scoring may affect participants performance. Standardization of stimuli and methods of analyses will assist in internal comparability between studies that use similar tasks. Standardization is a requirement for basic experimental control to minimize biased results and the influence of extraneous variables on participants’ performance (Fischer and Milfont 2010). To further understand age-related differences in pseudoneglect within each task discussed, standardization of instructions, administration, and scoring will be imperative. Similar to the administration of standardized psychometric testing, researchers examining pseudoneglect may benefit from the development of and standardization of visuospatial tasks. Standardized stimuli, administration, and scoring would assist in enhancing the internal comparability between studies, and the validity and reliability of the results obtained from testing.

Furthermore, the heterogeneity among the types of tasks used to assess pseudoneglect, including differences in task demands, decreases comparability between tasks. Previous research has failed to find evidence for the inter-task reliability of the line bisection, landmark, greyscales, and lateralized visual detection tasks, and this is proposed to result from differences in task demands (Learmonth et al. 2015a; Rueckert et al. 2002). The line bisection and landmark task have been considered to rely on *global size judgment* as both tasks involve assessing the midpoint along a horizontal line, whereas the greyscales task involves a *luminance judgment* and the lateral visual detection task involves a *stimulus detection* (Learmonth et al. 2015a). Lateralized spatial biases may be task-dependent and assumptions of equivalence in future reviews may be counterproductive.

However, it is also conceivable that improvements in research design, including smaller age ranges, screening for cognitive impairment, and standardization of tasks, may improve internal comparability, but may not improve reported inconsistencies. If the present inconsistencies in research examining age-related changes in pseudoneglect prove robust to improvements in research methodology, the field may find it necessary to acknowledge this pattern within the results and critically consider the validity of the findings. As such, given the variability in the conclusions reported by the studies included in the current review, the visuospatial tasks examined may not provide valid or reliable estimates of age-related changes in cognitive functioning. Specifically, the tasks included in the current review may not be sensitive enough to reliably differentiate the magnitude of pseudoneglect demonstrated by younger and older adults. Rather, the tasks may provide the greatest utility to clinicians when examining patients with brain injuries to assess for larger systematic biases, such as hemispatial neglect.

### Limitations

Arguably, a main limitation of this systematic review is the search strategy and eligibility criteria employed. One might have used additional keys words or subject headings to identify the “situation” (i.e., pseudoneglect). However, search terms used in the current study were identified in collaboration with a university librarian and content expert (LE) to enhance the identification of relevant articles. One might have also employed an alternative search strategy. For example, one could have conducted an additional search for studies involving pseudoneglect, regardless of the “population” (i.e., older adults), and subsequently screened for studies examining participants over the age of 60. Following the search employed in the current study and a search using only the “situation”, the studies selected from both searches could be compared to each other to ensure that the search was inclusive. Although this approach was not employed in the current study, forward and backward searching was conducted to enhance the likelihood that the search was exhaustive. Furthermore, with regard to the eligibility criteria, a limitation of the study is that the titles and abstracts were screened and the relevant articles were examined for eligibility by one author (TF); however, if there was doubt regarding whether to include or exclude an article during abstract screening, the article was included for full-text screening.

Another potential limitation of this review is the chance of publication bias. Overall, a large number of studies retrieved and included in the review (10) reported statistical comparisons between age groups that did not reach significance. Thus, there is a high chance that a number of other completed studies may not have been published due to inconclusive results. In an attempt to minimize this bias, grey literature was included in the review. Further, a large number of search terms that are synonymous with pseudoneglect were used and an inclusive inclusion criterion was used to screen for articles to allow for a broader and comprehensive overview of the research that included grey literature.

## Conclusion

Research to-date has reported an inconsistent relationship between aging and pseudoneglect. A number of recommendations for future research have been outlined throughout the review to enhance the field’s understanding. These include using smaller age ranges within age groups and differentiating between neurologically healthy participants and those with clinical diagnoses in an attempt to minimize the variability of spatial biases demonstrated by older adults; continued examination of gender to further investigate gender-specific aging effects; consistent use of stimuli and methods of analyses within each task to improve internal comparability; and, given limited inter-task reliability between the tasks included in the review, conduct future reviews by examining studies within tasks. Based on current evidence, although some age-related trends in visuospatial bias can be identified within each task, no firm conclusions about the effects of age on pseudoneglect can be drawn.

## Appendix 1

**Table:**

MEDLINE (OvidSP)	
1. older adult.mp. (4457)	
2. exp. AGING/ (225303)	
3. senior*.mp. (29084)	
4. exp. Adult Development/ (0)	
5. exp. Age Differences/ (0)	
6. elder*.mp. (208436)	
7. 1 or 2 or 3 or 4 or 5 or 6 (435635)	
8. pseudoneglect.mp. (184)	
9. exp. cerebral dominance/ not exp. language/ (64028)	
10. “hemispatial neglect”.mp. (435)	
11. hemineglect.mp. (313)	
12. “spatial bias”.mp. (297)	
13. laterali?ation.mp. not exp. language/ (6586)	
14. “visuospatial attention”.mp. (567)	
15. “spatial attention”.mp. (2681)	
16. exp. Visual Attention/ (0)	
17. “leftward bias”.mp. (198)	
18. “hemispheric asymmetry”.mp. not exp. language/ (1088)	
19. “hemispheric speciali?ation”.mp. not exp. language/ (707)	
20. 8 or 9 or 10 or 11 or 12 or 13 or 14 or 15 or 16 or 17 or 18 or 19 (70357)	
21. 7 and 20 (2038)	
22. limit 21 to (35english and human) (1524)	
